# Spatial Profiling of Germinal Centers in Mouse Secondary Lymphoid Organs Using MACSima Imaging Cyclic Staining Technology

**DOI:** 10.1002/eji.70157

**Published:** 2026-02-24

**Authors:** Isabel San Martín Molina, Hanneke Okkenhaug, Simon Walker, Michelle Linterman, Edith Marcial‐Juárez

**Affiliations:** ^1^ Imaging Facility, Babraham Institute Babraham Research Campus Cambridge UK; ^2^ Immunology Program, Babraham Institute Babraham Research Campus Cambridge UK; ^3^ Malaghan Institute of Medical Research Wellington New Zealand

**Keywords:** B cells, follicular dendritic cells, germinal centers, MACSima imaging cyclic staining, T follicular helper cells

## Abstract

We developed a 50‐plex imaging protocol using the MACSima platform to characterize the microarchitecture of germinal centers in secondary lymphoid tissues. This workflow combines tissue processing, automated cyclic imaging, image preprocessing, and a new analysis method that detects morphological changes in irregularly shaped stromal cells.

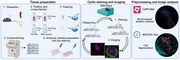

Germinal centers (GCs) form in secondary lymphoid organs (SLOs), such as lymph nodes (LNs), spleen, and tonsils, upon infection or vaccination. GCs give rise to antibody‐secreting cells and memory B cells that have diversified and improved their B cell receptors. When B cells encounter foreign antigens within highly compartmentalized environments in SLO, they undergo significant proliferation and somatic hypermutation, resulting in the formation of the GC dark zone. Subsequently, B cell clones with increased affinity to the antigen are selected by follicular dendritic cells (FDCs) and receive survival cues from T follicular helper (Tfh) cells in the light zone [1]. The study of the GC in its native microenvironment has advanced our core understanding of GC B cells and Tfh cells dynamics, and their interactions with antigens and other immune and stromal cells [2, 3]. This also enabled the discovery of localization defects in Tfh cells associated with ageing, deciphering mechanisms driving immunopathology of infectious diseases, and autoimmunity [4, 5, 6, 7].

MACSima Imaging Cyclic Staining (MICS) platform is a fluorescence‐based, automated system that operates in three cyclic steps: tissue staining, imaging acquisition, and photobleaching of the fluorescent signal (Figure 1A) [8, 9, 10]. Here, we designed and optimized a multiplex protein panel to deeply characterize GC cell populations in mouse spleen and LNs using MICS.

**FIGURE 1:**
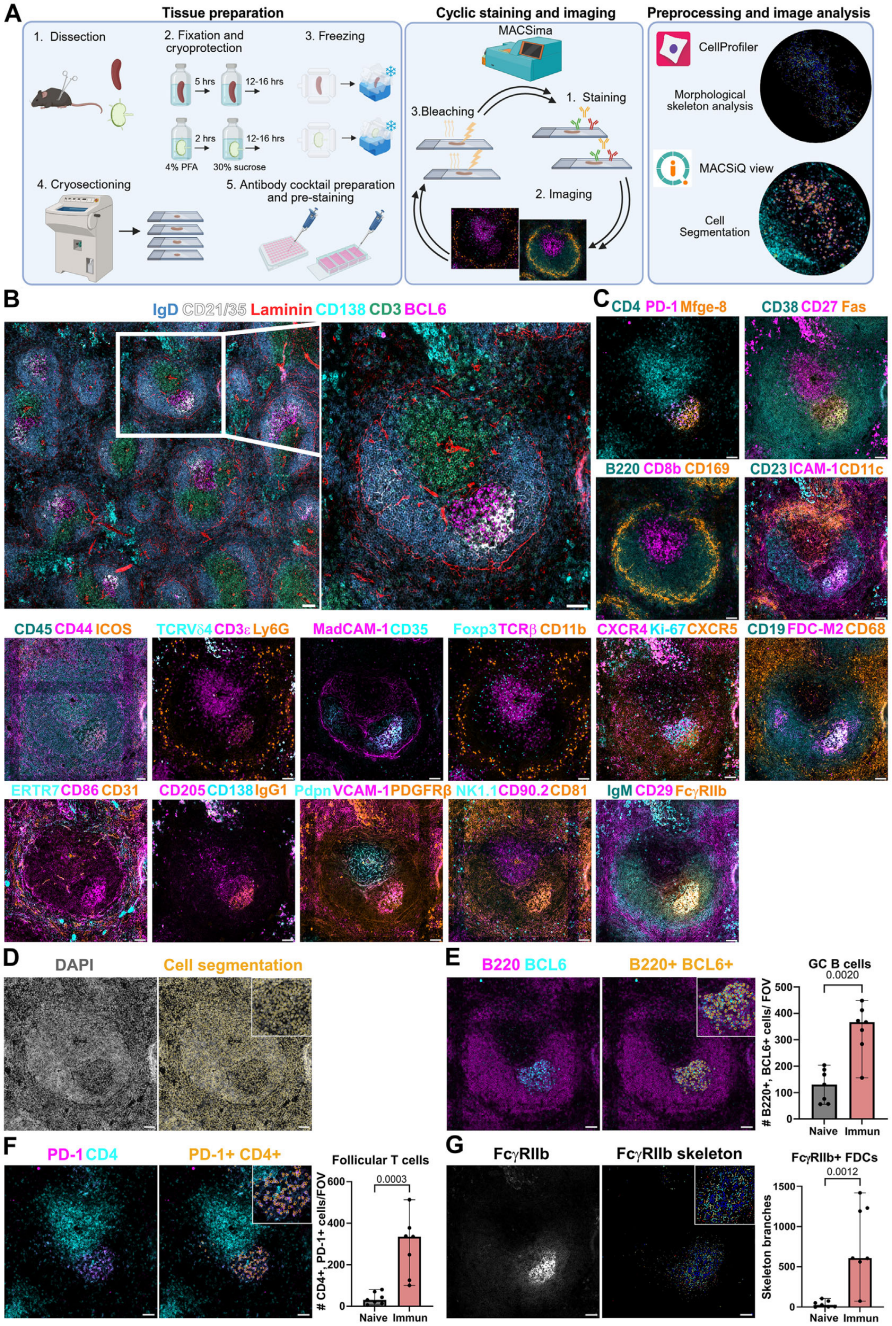
High‐plex imaging of spleen from NP‐KLH immunized mice and phenotypic analysis. (A) Workflow diagram. (B) Overview image and higher magnification image containing a GC in the spleen. (C) Phenotypic characterization of stromal and immune cells in the splenic white pulp. (D) Cell segmentation using nuclear staining. (E, F) Phenotypic analysis of GC B cells (B220+, BCL6+), and follicular T cells (PD‐1+, CD4+). (G) Morphological skeleton analysis of FcɣRIIb+ (CD16/32) FDC networks. Each dot in the bar graphs represents the quantification of independent fields of view (FOVs; 650 µm × 650 µm). Statistically significant *p* values (*p* < 0.05; one‐sided Mann–Whitney *U* test). Scale bars: 500 µm in B, and 300 µm in B (right) and C.

We immunized mice with a model antigen, 4‐hydroxy‐3‐nitrophenylacetyl (NP)‐keyhole limpet hemocyanin (KLH), which induces a T‐cell‐dependent GC response. We collected spleens and LNs 10 days after immunization, fixed them in 4% paraformaldehyde, cryoprotected, and froze them. Thin cryosections (6 µm) were collected to fit within a MACSwell frame (Figure S1A). First, we performed indirect immunofluorescence staining for MadCAM‐1, followed by direct immunofluorescence staining for Ki‐67, PD‐1, and DAPI. Panoramic views of Ki‐67 were obtained at 2× magnification to select regions of interest (ROIs) containing GCs (Figure S1A), and the focus was manually determined using DAPI staining with a 20× objective (Figure S2D,E). The three pre‐stained markers were first imaged, followed by 34 iterative cycles using the automated MICS (Table S1).

Image pre‐processing was performed using the integrated MACSiQ View software, which involved stitching together individual neighboring images, automatically determining optimal exposure times, and subtracting any residual fluorescent signal. We optimized the detection of proteins that facilitate the phenotypic characterization of multiple populations such as: GC B cells, light zone GC B cells, dark zone GC B cells, IgG1+ class‐switched GC B cells, antigen (NP)‐specific GC B cells (Figure S2A), Tfh cells, T follicular regulatory cells, tingible body macrophages, and plasma cells (Figure S2A, Table S2). FDCs are stromal cells essential for GC polarization and for trapping foreign antigens. Here, we identified FDCs with classical markers and characterized their phenotype in steady state and upon activation. We also identified other stromal cells involved in orchestrating the migration of immune cells within the SLOs including fibroblastic reticular cells, marginal reticular cells, lymphatic endothelial cells, and blood endothelial cells. Additionally, we evaluated other innate populations, including γδ T cells, natural killer cells, pan‐macrophages, metallophilic macrophages in spleen or subcapsular sinus macrophages in lymph nodes, conventional dendritic cells, and myeloid cells (Table S2; Figures 1B and 2A). This protocol is compatible for visualizing endogenously expressed red fluorescent protein (RFP) in genetically modified mice (Figure S2B) [11], and identifying donor cells from the host in adoptive transferred experiments by CD45.1 allotype expression (Figure S2C). We quantified the abundance of three main cell types involved in the GC: GC B cells, follicular T cells, and FDCs [12]. From a panoramic view of the entire LN, three to five ROIs of follicular areas were defined for quantification. Cell segmentation was performed using MACSiQ view software, which combined nuclear and membrane markers to accurately identify lymphocytes (Figures 1D and 2C). GC B cells were identified by the expression of B220 and BCL6 (Figure S3A) or Fas and CD19 (Figure S3A–C). Subcutaneous immunization with NP‐KLH induced a significant increase in the number of GC B cells in the spleen (Figure 1E) and in LNs (Figure 2D) compared with control mice (Figures S4A,B and S5A,B). Follicular T cells were identified based on the co‐expression of CD4 and PD‐1 (Figure S3A). We also observed a significant increase in the number of follicular T cells in spleens (Figure 1F) and LNs (Figure 2E) after immunization (Figures S4B and S5B).

**FIGURE 2:**
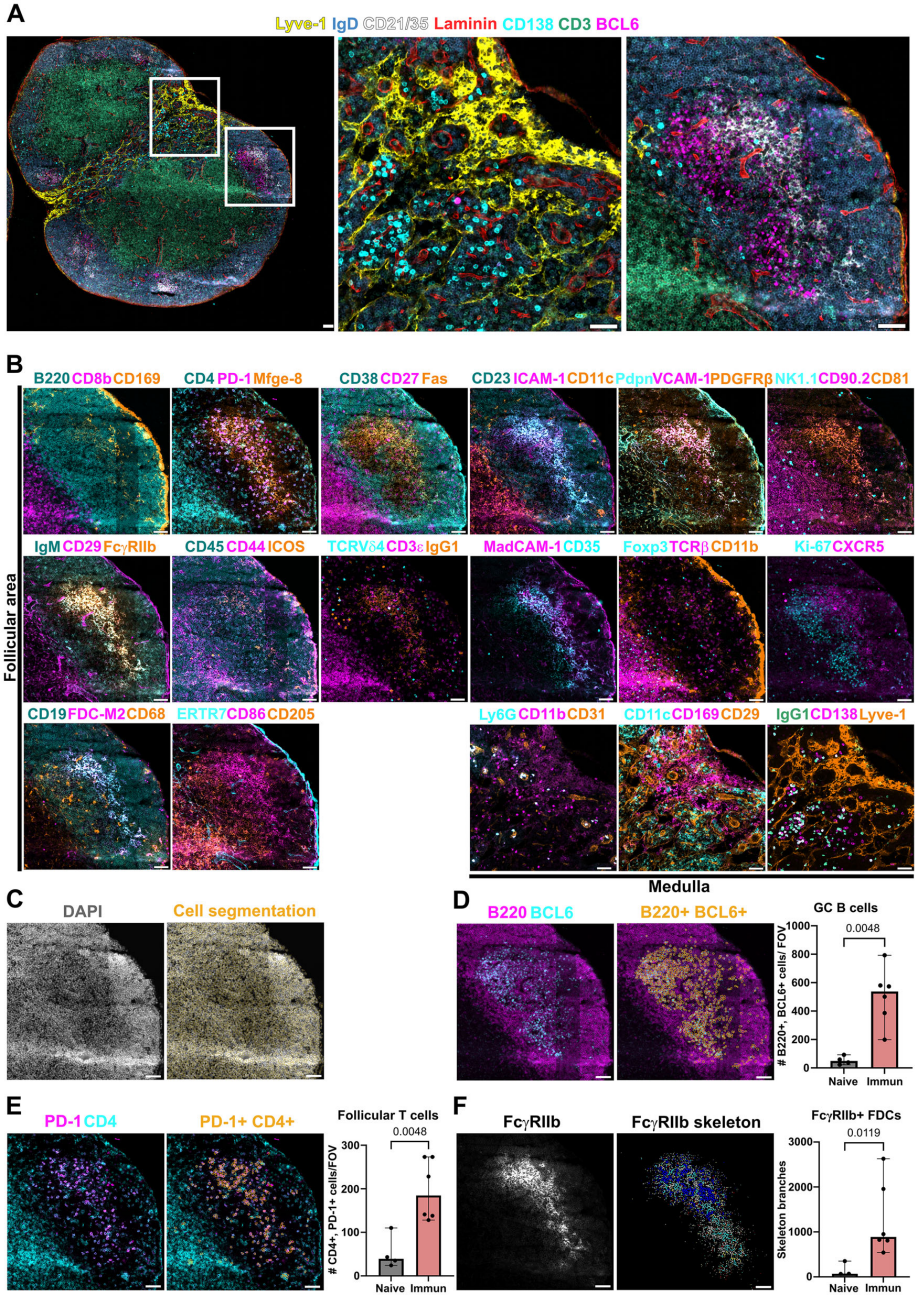
High‐plex imaging of mouse lymph nodes from NP‐KLH immunized mice and phenotypic analysis. (A) Panoramic overview of a LN and higher magnification images of the medulla (middle) and a GC within the follicle (right). (B) Phenotypic characterization of stromal and immune cells in LN. (C) Segmented cells outlined in yellow in the follicular area. (D, E) Phenotypic analysis of GC B cells (B220+, BCL6+), and follicular T cells (PD1+, CD4+). (F) Morphological skeleton analysis of FcɣRIIb+ (CD16/32) FDC networks. Each dot in the bar graphs represents the quantification of an independent FOVs (500 µm × 500 µm). Statistically significant *p* values (*p* < 0.05; one‐sided Mann–Whitney *U* test). Scale bars: 500 µm in A (left), and 300 µm in A (middle and right) and B.

Single‐cell phenotyping using MACSiQ view software was suitable for quantifying GC B cells and follicular T cells. However, FDCs form a mesh‐like network in SLO and expand upon immunization by spreading their membranes between cells, resulting in a reticular pattern. Single‐cell segmentation performed in MACSiQ view software recognized adjacent cells to FDCs, but not FDCs themselves (Figure S6A). Skeleton analysis has been commonly used to assess morphological changes in irregularly shaped cells such as neurons [13]. Here, we implemented it to capture topological changes in FDC networks. We designed a CellProfiler pipeline using CD21/35 (Figure S6B) and FcɣRIIb (CD16/32; Figure S6C) images to analyze FDC network morphology. We found that this new quantification approach outperformed standard single‐cell segmentation for the detection of FDC network expansion after immunization in spleens (Figure 1G) and LNs (Figure 2F).

In summary, we optimized an extensive proteomic panel for analyzing innate and adaptive immune cells in mice, with an emphasis on GC biology, using MICS platform. It utilizes off‐the‐shelf, directly conjugated antibodies; however, the performance of antibodies can differ depending on the tissue of interest and the preservation method used. Therefore, it is essential to validate and optimize antibody dilutions, incubation and bleaching times, and imaging acquisition parameters. This can be time‐consuming and expensive but ensures optimal signal‐to‐noise ratios and minimizes residual signal between cycles. MICS technology relies on direct immunofluorescence, which makes it challenging to detect low intensity expressed proteins. Here, we limited the use of indirect immunofluorescence for detecting MadCAM‐1 and Mfge‐8 by using an anti‐rat or anti‐hamster secondary antibody, respectively. This technology has the potential to image more than a hundred protein biomarkers while the tissue remains intact; therefore, tissue integrity is of high priority. We found paraformaldehyde‐fixed frozen tissue is more resilient than fresh‐frozen tissue, ensuring consistent antibody performance. MICS platform can image large areas on a standard microscopy slide, resulting in multiple days of acquisition, which generates terabytes of data. Additionally, the analysis of high‐content, complex imaging data relies on expensive licensed software that is limited to a single computer. Bioimaging facilities must consider data storage capabilities and budget when adopting this technology. Lastly, traditional intensity‐based segmentation methods, such as the integrated into the MACSiQ View software, are designed to quantify round‐shaped cells. Therefore, the quantification of irregularly shaped cells requires independent processing using alternative open‐source software.

## Conflicts of Interest

Dr Michelle Linterman declares funding from GSK outside of this work. The other authors declare no conflicts of interest.

## Supporting information




**Supporting File**: eji70157‐sup‐0001‐SupMat.pdf.

## Data Availability

The data that supports the findings of this study are available from the corresponding authors upon reasonable request.
