# Supplementary material for: Spatial Profiling of Germinal Centers in Mouse Secondary Lymphoid Organs Using MACSima Imaging Cyclic Staining Technology
Source: Eur J Immunol. 2026 Feb 24;56(2):e70157. doi: 10.1002/eji.70157 (PMC12930330; doi:10.1002/eji.70157)
Supplement: Supplementary file 1 — Supporting File: eji70157‐sup‐0001‐SupMat.pdf. [file EJI-56-e70157-s001.pdf]

Supplementary information

Materials and methods

Mouse maintenance, husbandry and immunizations

C57BL/6, OT-II TCR-Tg, *Il21*$^{Cre/+}$ *Rosa26*$^{+/iRfp}$, Cd4$^{Cre/+}$ Cxcr5$^{fl/fl}$ mice were bred and housed in the Babraham Institute Biological Support Unit. The Babraham Institute Animal Welfare and Ethical Review Body approved all mouse experimentation. All procedures were performed in accordance with the Home Office legislation for animal research in the United Kingdom (PPL: PP9973990). Mice were subcutaneously immunized in both flanks with 50 µg of NP-KLH (N-5060-25, Biosearch Technologies) in Alhydrogel (vac-alu-10, Invivogen) or intraperitoneally with 100 µg of NP-KLH in Alhydrogel. Animals in the control group were injected with phosphate-buffered saline (PBS).

Specimens and tissue processing for multiplex imaging

Ten days after the immunizations, inguinal LNs and spleens were dissected, the surrounding fat was removed, and tissues were collected in cold PBS. Spleens were fixed in 4% paraformaldehyde (J19943-K2, ThermoFisher Scientific) for 5 hours, and lymph nodes were fixed for 2 hours at 4°C. The tissues were washed in PBS for 20 minutes and incubated in 30% sucrose (11377968, Fisher Scientific) overnight. Custom aluminum foil molds were prepared by shaping aluminum foil around a square-shaped cuvette and filled with optimal cutting temperature compound (AGR1180, Agar Scientific). Five to seven LNs were aligned to the bottom of the mold, snap-frozen, and stored at –80°C. Spleens were individually placed in aluminum foil, snap-frozen, and sealed securely. Six-micrometer cryosections were obtained using a Leica CM3050 S Cryostat set up to a temperature between –15°C and –18°C, collected in 1 mm SuperFrost Plus glass slides (631-0108, VWR), and stored at -20°C. Tissue slides were thawed and air-dried for 30 minutes at room temperature, mounted in the MACSwell™ one imaging frame (130-124-673, Miltenyi Biotec) and rehydrated with MACSima™ running buffer (130-121-565, Miltenyi Biotec). Nonspecific binding was prevented by incubating the tissues with blocking buffer containing 10% normal donkey serum (D9663, Sigma Aldrich) and 2% bovine serum albumin (A9647, Sigma Aldrich) in PBS for 45 minutes. Subsequently, cryosections were incubated with MadCAM-1 antibody (MECA-367) for 1 hour, followed by Alexa Fluor 555-conjugated anti-Rat IgG for 30 minutes (primary and secondary antibody details are described in **Table S1**). After washing, free binding sites to rat immunoglobulin were blocked with 20% normal rat serum (10710C, ThermoFisher Scientific) in 2% bovine serum albumin for 45 minutes. Ki-67 (REA183) and PD-1 (RMP1-30) antibodies were incubated for 1 hour at room temperature (**Table S1**). Finally, DAPI staining was performed for 10 minutes, and slides were washed three times (130-127-575, Miltenyi Biotec). A set volume of MACSima™ running buffer was added to the MACSwell™ imaging frame according to the well size (475 µL in a four-well frame, 950 µL in a two-well frame, 1900 µL in a one-well frame). The automated needle pipettes out the buffer once the carrier is mounted, before proceeding

with the automated cyclic staining. Information on antibodies used in this experimental procedure is listed in **Table S1**, protein markers chosen to identify the different cell populations in the high-plex panel are listed in **Table S2**, and the materials and reagents used in this work are listed in **Table S3.**

Multicycle imaging using MICS imaging platform

The abundance and localization of the biomarkers guided the panel design. Antibodies targeting low-abundance and intracellular markers were incubated for 30 minutes and imaged in the initial cycles. Highly expressed membrane markers were assigned to later cycles and incubated for 10 minutes (**Table S1**). Antibody performance was previously assessed by evaluating the distinct spatial expression patterns of target markers and the signal-to-noise ratio using single-plex confocal imaging. Automated cyclic immunofluorescence was performed using an LED-calibrated MACSima™ platform (control module v1.1.0) incorporating thirty-four iterative imaging cycles. The signal between imaging cycles was removed by photobleaching. A cocktail with three different antibodies per cycle and DAPI (130-127-575, Miltenyi Biotec) was prepared in a 96-deepwell plate (130-126-865, Miltenyi Biotec) and loaded simultaneously with the carrier containing the tissue. The system sequentially accesses the designated wells during the automated acquisition and transfers the corresponding antibody cocktail to the tissue in each cycle. Tissues were re-stained with DAPI every eight cycles. If DAPI staining occurred in a 10-minute incubation cycle, it was diluted 1:50; if it occurred in a 30-minute incubation cycle, it was diluted 1:200. To remove unbound antibodies after incubation, washes with MACSima™ running buffer were performed automatically between cycles. Single-color images were captured using sensitivity mode, and images with two exposure times, “high” and “low”, corresponding to a 1:8 duration ratio, were acquired. Exposure levels were defined as percentages, with 100% corresponding to a standard exposure time of 50 ms for each fluorophore. This resulted in minimum exposure times of 149 ms and maximum exposure times of 2,500 ms. Higher percentages reflect longer acquisition times to capture dim signals and vice versa. At the pre-processing stage, the software selects the image with the optimal exposure settings. We used an average exposure level of 100% for APC/Alexa Fluor 647, 635% for PE/Cy3/Alexa Fluor 555, and 500% for FITC/Alexa Fluor 488. The DAPI exposure setting was consistently set at 100%, corresponding to an exposure time of 50 ms. The average photobleaching durations were 6 minutes for APC/Alexa Fluor 647 (470 J), 4 minutes for PE/Cy3/Alexa Fluor 555 (600 J), and 3 minutes for FITC/Alexa Fluor 488 (300 J). A panoramic view of the entire slide with the tissues was obtained with the 2× objective with a numerical aperture of 0.1 on the integrated inverted widefield epifluorescence microscope. Regions of interest (ROIs) were selected in areas containing GCs identified as clusters of Ki-67+ cells (**Figure S1A**). The focus was defined by manually setting a constant z-offset based on the DAPI signal. High-resolution images were acquired using the integrated monochromatic scientific CMOS camera (resolution: 0.170 µm/pixel) with the 20× objective and a numerical aperture of 0.45, on standard 1 mm glass slides. For the simultaneous acquisition of spleen and LNs samples, we used a MACSwell™

one imaging frame (130-124-673, Miltenyi Biotec). Multiple ROIs containing up to 30 FOVs were imaged, and the acquisition of the experiment lasted approximately nine days. We also assess the integrity of the tissue from cycle one to thirty-four by staining with DAPI. The nuclear morphology was maintained despite multiple cycles of washing and photobleaching (**Figure S2D, E**).

Image processing and analysis using MACSiQ View software

Image processing steps included flatfield and distortion correction, cycle registration, stitching, background subtraction, and camera noise correction, as previously described [9]. Raw image data (OME-TIFF files) were processed using MACSiQ software (v1.3.2). We applied a standardized pipeline designed to correct these image artefacts and subtract acquired background images from captured signal images. Image files were initially converted from 16-bit to 32-bit for computation and normalized back to 16-bit post-processing. Optimal exposure times are automatically determined by the best fit on a high-dynamic range image obtained by capturing two images with different exposure times per marker. Additionally, any residual fluorescent signal from the previous cycle that remained after photobleaching was subtracted. Camera noise was removed using calibration files, and flatfield correction was applied using lens- and channel-specific references. Chromatic and lens distortions were corrected based on sub-pixel calibration data. The DAPI channel is set as the reference for aligning and co-registering all imaging channels in the full-plex panel. Images captured previously to the next staining step were used for background subtraction, and a final normalization applied a 99.9999th percentile threshold to rescale boosted pixels. When large areas including multiple tiles were captured, like in the case of entire LNs or multiple white pulp regions in the spleen, stitching was applied on the software itself. Stitched images were blended using a 200-pixel Gaussian overlap, cropped to DAPI (DAPI image from the first cycle), and scaled to 50% (0.34 µm/pixel to improve image segmentation and reduce data size. Processed images were saved to the output folder and opened using the MACSiQ View software for visualization and analysis.

Cell-segmentation was performed using the MACSiQ View software to quantify total cell counts in 16-bit processed images (0.34 µm/pixel). For quantification of GC B cells and follicular T cells, we selected a set size (500 µm × 500 µm) ROIs in follicular areas for analysis of the LNs. We selected square ROIs (650 µm × 650 µm) of the white pulp for spleen samples. From a panoramic view of the entire LN, three to five ROIs of follicular areas were defined for quantification. Cell segmentation was performed independently for each ROI, combining nuclear and cytoplasmic parameters. We employed the advanced morphology tissue segmentation algorithm based on the DAPI signal for nuclear segmentation. For LNs, nuclear segmentation parameters included: nucleus size of 10–50 pixels, detection sensitivity of 120, separation force of 80, and sigma smoothing of 1.0. For spleens, the nucleus size range was set to 10–40 pixels with the same detection sensitivity, and a separation force of 70. Cytoplasmic segmentation was conducted using the constrained doughnut method with B220

(CD45R) and CD45 as marker inputs, with a doughnut width of 20 pixels and a sensitivity setting of 100 for LN analysis. Identical cytoplasmic segmentation parameters were used for segmentation in spleen samples, except for a doughnut width of 15 pixels. Quantification of follicular T cells ($CD4^+$, $PD1^+$), GC B cells ($B220^+$, $BCL6^+$ or Fas^+, $CD19^+$), and FDCs ($CD16/32^+$, $CD21/35^+$) was defined through marker-based gating analysis using the Workflow Editor (**Figure S3A; Figure S6A**). Additionally, individual channels, CD16/32 and CD21/35, for the corresponding FOVs were exported as TIFF files for independent analysis of FDC networks in CellProfiler (**Figure S6B, C**).

Image analysis using CellProfiler

To evaluate topological changes in FDC, we employed a skeleton-based quantification method that has been used for irregularly shaped cells, such as neurons. Single-channel CD21/35 and CD16/32 TIFF images were analyzed using CellProfiler (v4.2.8). First, we used an image enhancement and background subtraction step utilizing *EnhanceOrSuppressFeatures* (feature type set to "speckles" and a size to 10, and processing set to fast speed and accuracy). This step improved skeleton definition and reduced background noise. Subsequently, a grayscale input image was converted into a binary image using manual thresholding (*Threshold* module, with a threshold value of 0.1 and no smoothing). Next, we used the *MorphologicalSkeleton* module to generate a skeletonized version of the image. Several morphological parameters in the resulting skeleton were measured using the *MeasureImageSkeleton* module, including the number of skeletons, the number of endpoints (defined as terminal nodes of skeleton branches not connected to any other segment), and the number of branches (defined as slab segments connecting endpoints and junctions). The resulting skeleton image was converted into an object by using the *IdentifyPrimaryObjects* module. Finally, we utilized the *MeasureObjectIntensity* module to measure the intensity values within the skeleton object, which was used to calculate the total length of the skeleton (**Figure 1G; Figure 2F; Figure S6B, C**).

Statistical analysis

All analyses and data graphs were generated using GraphPad Prism (version 10.3.0). We performed a one-sided *Mann-Whitney U* test to evaluate statistically significant changes between experimental groups in both LNs and spleens. Data in graphs are presented as median with 95% confidence intervals, and the thresholding for statistical significance was defined as $p < 0.05$.

Supplementary Tables

Table S1. High-plex 50-marker panel for characterizing germinal centers in mouse lymph nodes and spleens.

Antibody	Clone	Catalog number	Supplier	Fluorochrome	Incubation time	Dilution factor 1:x
BCL6	REA373|7D1, IG191E/A8	130-121-997, 648306	Miltenyi Biotec, Biolegend	APC, Alexa Fluor AF647	30 minutes	50,50
CD3	17A2	100236	Biolegend	APC	30 minutes	50
CD3	REA641|17A2	130-121-133	Miltenyi Biotec	PE	30 minutes	50
CD3epsilon	145-2C11	130-102-792	Miltenyi Biotec	PE	10 minutes	50
CD4	REA604|RM4-5	130-116-487	Miltenyi Biotec	APC	30 minutes	70
CD8b	REA793|H35-17.2	130-111-634	Miltenyi Biotec	APC	30 minutes	70
CD11b	REA592|M1/70.15.11.5	130-113-806	Miltenyi Biotec	PE	30 minutes	50
CD11c	N418	117310	Biolegend	APC	30 minutes	50
CD16/CD32 (FcgrIII/FcgrII)	2.4G2	553145	BD Bioscience	PE	30 minutes	100
CD19	REA749| 6D5	130-111-883	Miltenyi Biotec	PE	30 minutes	50
CD21/35	REA800|7E9	130-111-729	Miltenyi Biotec	FITC	30 minutes	100
CD23	B3B4	130-102-611	Miltenyi Biotec	PE	30 minutes	50
CD27	REA499|LG.3A10	130-113-640	Miltenyi Biotec	PE	30 minutes	70
CD29	REA1074|HMß1-1	130-119-165	Miltenyi Biotec	PE	30 minutes	80
CD31	REAL260	130-115-988	Miltenyi Biotec	APC	10 minutes	50
CD35	8C12	558768	BD Biosciences	Alexa Fluor 488 (in-house conjugated)	30 minutes	100
CD38	90.4	130-103-030	Miltenyi Biotec	APC	30 minutes	70
CD44	IM7	17-0441-83	ThermoFisher Scientific	APC	10 minutes	70
CD45	REA737|30F11	130-110-658	Miltenyi Biotec	FITC	10 minutes	100
CD45.1	A20	110720	Biolegend	Alexa Fluor 647	30 minutes	100

CD45R (B220)	REA755|RA3-6B2	130-110-708	Miltenyi Biotec	FITC	10 minutes	100
CD54 (ICAM-1)	3E2	553253	BD Biosciences	PE	30 minutes	100
CD68	REA835|FA-11	130-112-856	Miltenyi Biotec	PE	30 minutes	70
CD81	EAT2	130-126-363	Miltenyi Biotec	FITC	10 minutes	80
CD86 (BT-2)	GL1	12-0862-82	ThermoFisher Scientific	PE	15 minutes	50
CD90.2	REA1167|30-H12	130-120-898	Miltenyi Biotec	APC	30 minutes	70
CD95 (FAS)	REA453|Jo2	130-123-568	Miltenyi Biotec	APC	30 minutes	70
CD106 (VCAM-1)	REA971|429	130-116-322	Miltenyi Biotec	FITC	30 minutes	100
CD138 (Syndecan-1)	REA104|281-2	130-122-945	Miltenyi Biotec	APC	30 minutes	80
CD140b (PDGFRβ)	APB5	130-123-271	Miltenyi Biotec	PE	30 minutes	50
CD169 (Siglec-1)	REA197|3D6.112	130-124-896	Miltenyi Biotec	APC	30 minutes	100
CD184 (CXCR4)	2B11|CXCR4	551966	BD Bioscience	PE	30 minutes	70
CD185 (CXCR5)	REA215|2G8	130-123-753	Miltenyi Biotec	PE	30 minutes	50
CD205 (DEC205)	NLDC-145	130-102-906	Miltenyi Biotec	PE	30 minutes	50
CD278 (ICOS)	C398.4A	313514	Biolegend	Alexa Fluor 488	30 minutes	70
CD279 (PD-1)	RMP1-30	109117	Biolegend	Alexa Fluor 647	60 minutes	100
Donkey a-rat Alexa Fluor 555	Polyclonal	712-565-153	Jackson ImmunoResearch	Alexa Fluor 555	30 minutes	1000
ER-TR7	ER-TR7	sc-73355	Santa Cruz	Alexa Fluor 488	15 minutes	150
FDC-M2	FDC-M2	212-MK-1FDCM2	Amsbio	Alexa Fluor 488 (in-house conjugated)	30 minutes	100
Goat a-Armenian hamster Cy3	Polyclonal	127-165-160	Jackson ImmunoResearch	Cy3	10 minutes	500
Foxp3	REA788|3G3, FJK-16s	130-111-679, 17-5773-82	Miltenyi Biotec, ThermoFisher Scientific	APC, APC	30 minutes	50, 50

IgD	REA772|11-26c.2a	130-111-497	Miltenyi Biotec	APC	30 minutes	80
IgG1	REA1017|X-56	130-117-099	Miltenyi Biotec	APC	15 minutes	50
IgM	REA979|X-54	130-116-311	Miltenyi Biotec	FITC	30 minutes	80
Ki-67	REA183|B56	130-117-691	Miltenyi Biotec	FITC	60 minutes	100
Laminin	Polyclonal	PA5-22901	ThermoFisher Scientific	DyLight 488	10 minutes	150
Ly6G	REA526|1A8	130-123-780	Miltenyi Biotec	PE	10 minutes	50
LYVE1	ALY7	53-0443-82	ThermoFisher Scientific	Alexa Fluor 488	20 minutes	80
MadCAM-1	MECA-367	120702	Biolegend	Unconjugated	60 minutes	100
MFGE8	18A2-G10	D199-3	MBL	Unconjugated	10 minutes	500
NK1.1	PK136	130-117-528	Miltenyi Biotec	APC	30 minutes	50
NP (29)	---	N-5070-1	Biosearch Technologies	PE	20 minutes	150
Podoplanin (gp38)	8.1.1	127410	Biolegend	APC	30 minutes	80
TCRβ	REA318|H57-597	130-123-842	Miltenyi Biotec	APC	30 minutes	50
TCRVδ4	REA372|GL2	130-105-722	Miltenyi Biotec	PE	30 minutes	40

Abbreviations: APC, allophycocyanin; CD, cluster of differentiation; Cy, cyanine; FITC, fluorescein isothiocyanate; Ig, Immunoglobulin; PE, Phycoerythrin; REA, Recombinant engineered antibody.

Table S2. Phenotypic characterization of immune and stromal cells in mouse spleen and LNs.

Cell populations	Biomarkers
**Lymphocytes**	
B cells	CD19<br>B220<br>IgD<br>IgM<br>CD38<br>CD23
Germinal center B cells	B220, BCL6<br>Fas, CD19
Antigen (NP)-specific GC B cells	B220, BCL6, NP
IgG1+ class-switched GC B cells	B220, BCL6, IgG1
Dark zone germinal center B cells	B220, BCL6, CD86-
Light zone germinal center B cells	B220, BCL6, CD86+
Plasma cells	CD138
T cells	CD3<br>TCRβ<br>CD4<br>CD8β<br>CD90.2<br>CD27
Regulatory T cells	CD4, Foxp3
T follicular helper cells	CD4+, PD-1+, Foxp3-
T follicular regulatory cells	CD4+, PD-1+, Foxp3+
Hematopoietic cells	CD45
Adoptive transferred cells	CD45.1
Cell activation	CD44<br>ICOS<br>CD86
Cell proliferation	Ki-67
Chemokine receptors	CXCR4<br>CXCR5
**Innate cells**	
Myeloid cells	CD11b<br>Ly6G
Natural killer cells	NK1.1
Conventional dendritic cells	CD11c<br>CD205
Metallophilic macrophages (spleen)/<br>Subcapsular sinus macrophages (LNs)	CD169
Pan-macrophages	CD68
Tingible body macrophages	CD68 (within GC area)
γδ T cells	CD3<br>TCRVδ4

Stromal cells	
Follicular dendritic cells	CD21/35<br>CD35<br>FcɣRIIb (CD16/32)<br>FDC-M2<br>Mfge-8<br>ICAM-1<br>CD23<br>VCAM-1
Fibroblastic reticular cells	ER-TR7<br>Podoplanin<br>Laminin<br>ICAM-1<br>VCAM-1
Marginal reticular cells	MadCAM-1
Blood endothelial cells	CD31
Lymphatic endothelial cells	Lyve-1
Red pulp/Perivascular stromal cells	CD140b (PDGFRβ)<br>CD29

Table S3. Key resources table

Reagents and materials	Catalog number	Supplier
Alhydrogel adjuvant 2%	vac-alu-10	InvivoGen
Alexa Fluor™ 488 Antibody Labeling Kit	A20181	ThermoFisher Scientific
Bovine Serum Albumin	A9647	Sigma Aldrich
4-Hydroxy-3-nitrophenylacetyl-Keyhole Limpet Hemocyanin (NP-KLH)	N-5060-25	Biosearch Technologies
Low-profile Microtome Blades	10687875	Fisher Scientific
MACSwell™ Deepwell Plates	130-126-865	Miltenyi Biotec
MACSwell™ Imaging Frames	130-124-676, 130-124-673, 130-126-794, 130-124-675	Miltenyi Biotec
MACSima™ Running Buffer	130-121-565	Miltenyi Biotec
MACSima™ Stain Support Kit, mouse	130-127-575	Miltenyi Biotec
MACSwell™ Sealing Foils	130-126-866	Miltenyi Biotec
MACSima™ System Buffer	130-125-315	Miltenyi Biotec
Normal donkey serum	D9663	Sigma Aldrich
Normal rat serum	10710C	Sigma Aldrich
Optimal cutting temperature Compound	AGR1180	Agar Scientific
Paraformaldehyde solution (4% in PBS)	J19943.K2	ThermoFisher Scientific
Sucrose	11377968	Fisher Scientific
Superfrost Plus slides	631-0108	VWR

Supplementary Figures

Figure S1

A

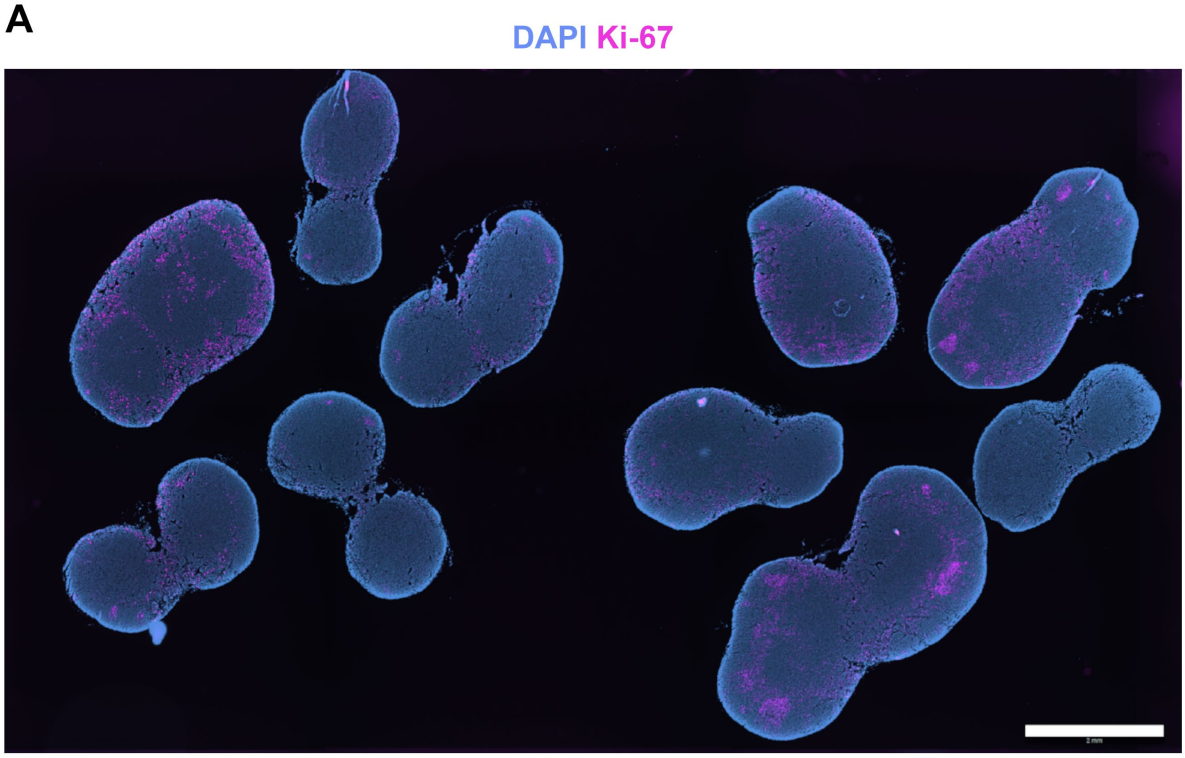

Figure S1. Representative overview of MACSwell™ imaging frame for imaging of lymph nodes. (**A**) Overview image of a single well containing ten LNs placed in a small area of 190 mm^2 (8x17 mm) of a four-MACSwell™ imaging frame. DAPI staining (light blue) and Ki-67 signal (magenta) were used to focus on the image and identify GCs, respectively. Scale bar: 2 mm.

Figure S2

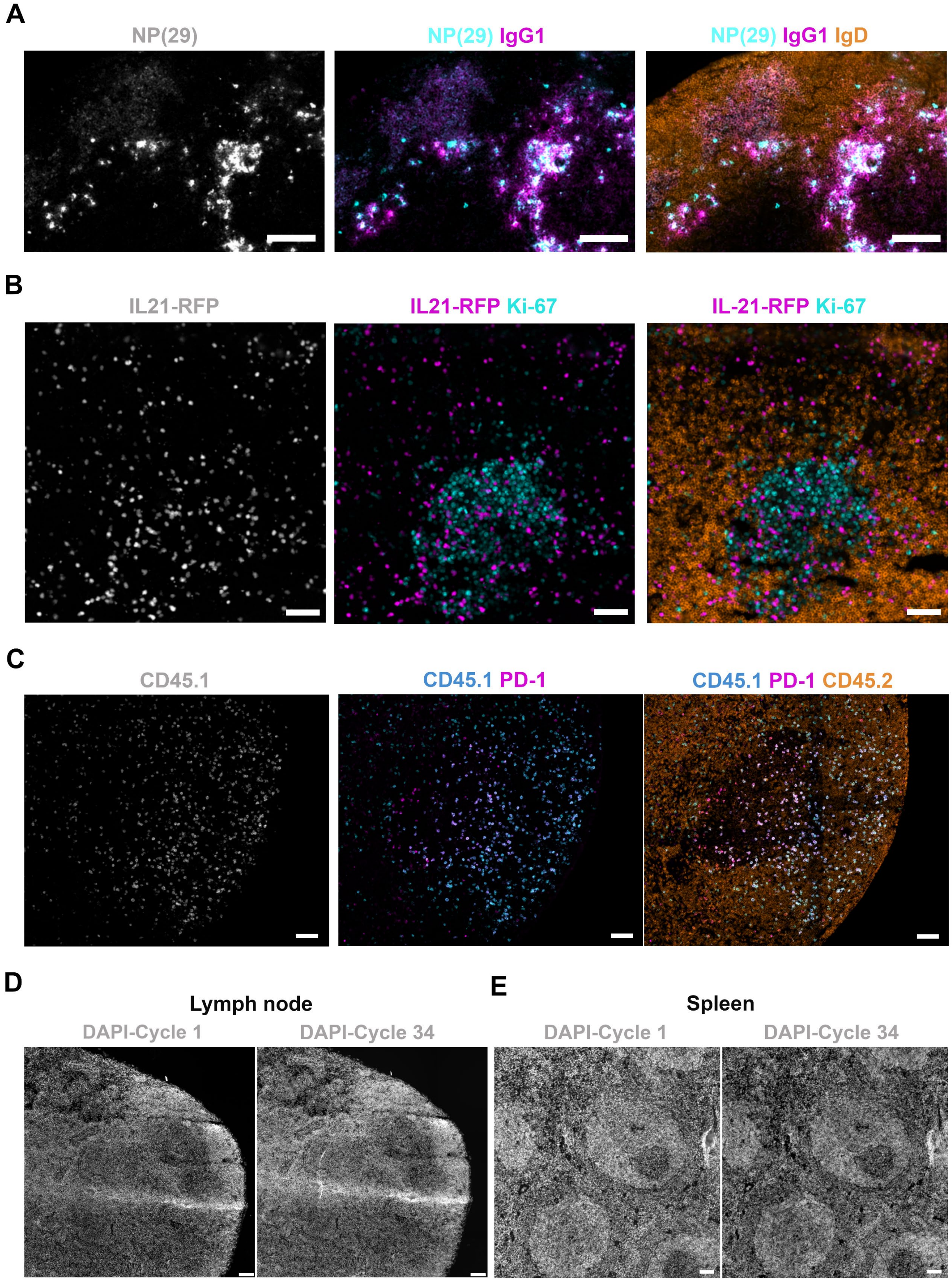

Figure S2. Illustrative images of lymph nodes showing endogenously expressed RFP, NP+ cells, CD45.1+ donor cells, and evaluation of tissue integrity. (**A**) Antigen (NP)-specific GC B cells and plasma cells in the LN of an NP-KLH immunized mouse. High-intensity IgG1+ and NP+ cells are characteristic of antigen-specific, class-switched plasma cells. IgD expression is shown

for reference to identify the follicular zone and GC. (**B**) IL-21+ fate-mapped cells in *Il21*$^{Cre/+}$*; Rosa26*$^{+/iRfp}$ mice. IL-21, Ki-67, and IgD merged images are displayed for visualization of RFP+ cells in the GC of the LN. (**C**) Images display OT-II donor-derived PD-1+ follicular T cells expressing the CD45.1 allele. They are distinguished from the host (Cd4$^{Cre/+}$ Cxcr5$^{fl/fl}$ mice) expressing the CD45.2 allele. (**D, E**) Images displaying DAPI staining in LNs (**D**) and spleen (**E**) were captured at cycle 1 and cycle 34 to evaluate tissue integrity. The nuclear morphology was maintained despite multiple cycles of washing and photobleaching. Scale bars: 300 µm. Abbreviations: CD, cluster of differentiation; Ig, Immunoglobulin; NP, 4-hydroxy-3-nitrophenylacetyl; RFP, red fluorescent protein.

Figure S3

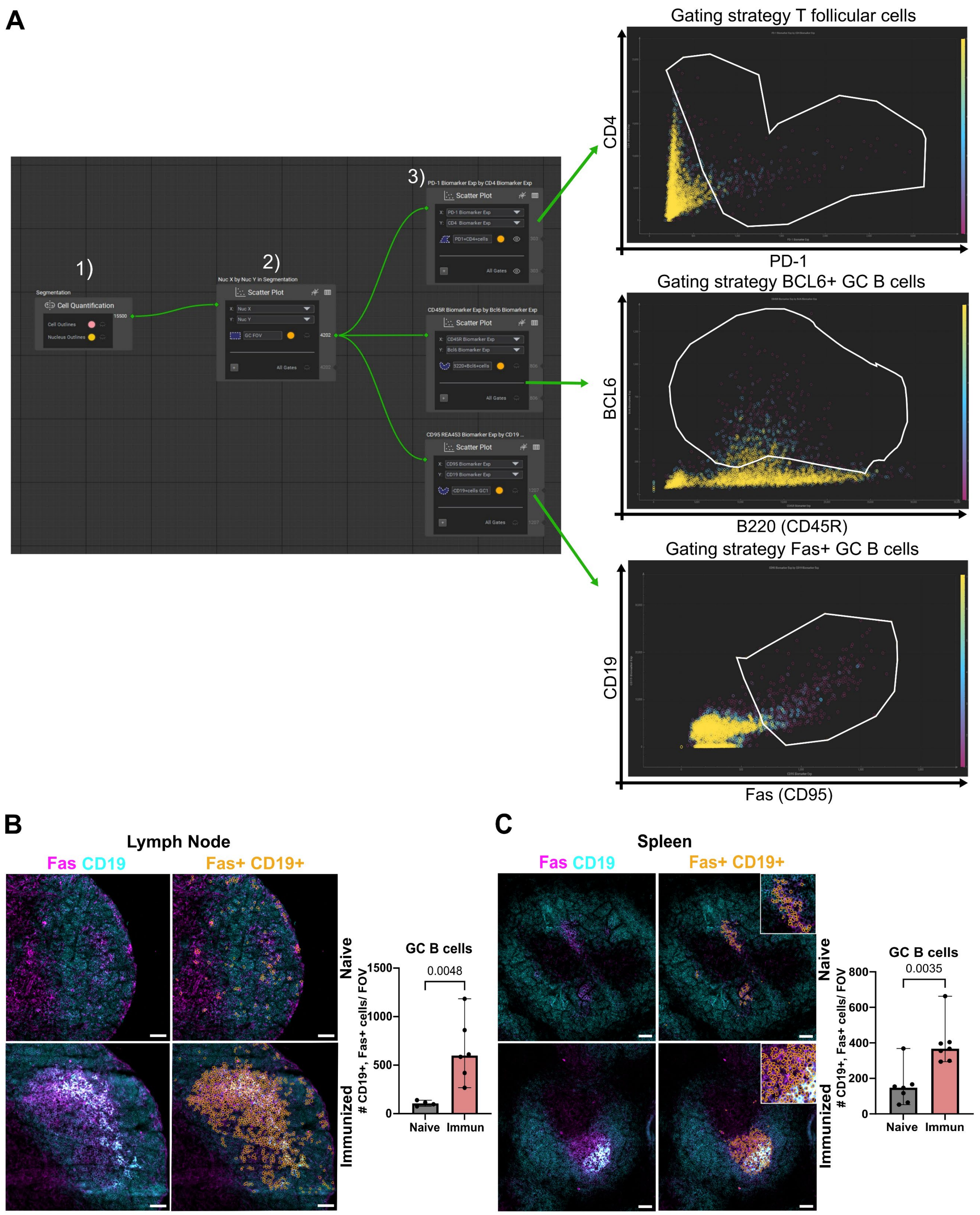

Figure S3. Gating strategy in the MACSiQ view software to identify GC B cells and follicular T cells. (**A**) MACSiQ view image analysis workflow showing 1) cell segmentation and quantification of total cells, 2) spatial localization of the selected FOV using X and Y coordinates, and 3) gating strategy in a two dimension dot plot graph to select CD4+, PD-1+ follicular T cells (upper panel), B220+, BCL6+ GC B cells (middle panel) and Fas+, CD19+ GC B

cells (bottom panel). GC B cells and follicular T cells were quantified in individual images with a size of 650 µm × 650 µm for the spleen, and 500 µm × 500 µm for the LN. (**B**, **C**) Reference images displaying Fas and CD19 double-positive cells outlined in orange for both control and immunized mice in LNs (**B**) and spleen (**C**). The corresponding graphs represent the number of GC B cells in immunized and control mice. Statistically significant *p* values ($p < 0.05$; one-sided *Mann-Whitney U test*). Scale bars: 300 µm. Abbreviations: CD, cluster of differentiation; GC, germinal center; FOV, field of view.

Figure S4

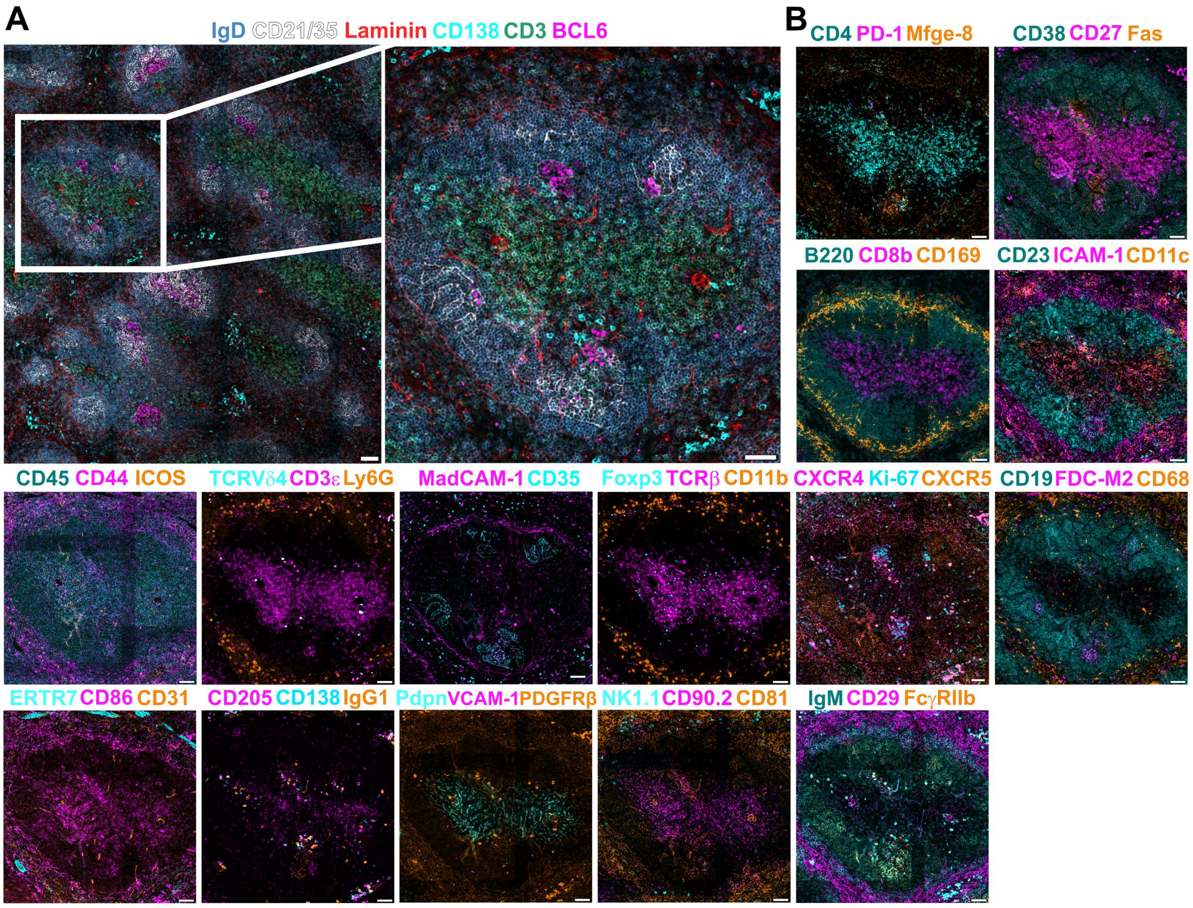

Figure S4. High-plex imaging of the spleen from a naive mouse. (**A**) Representative panoramic overview image of a spleen section from a control mouse showing six biomarkers. The white square corresponds to an individual white pulp area, which is shown at higher magnification. (**B**) Composite three-color combination photomicrographs show the different cellular markers. Scale bars: 500 µm for the overview image in panel A (left), and 300 µm for individual photomicrographs in panels A (right) and B.

Figure S5

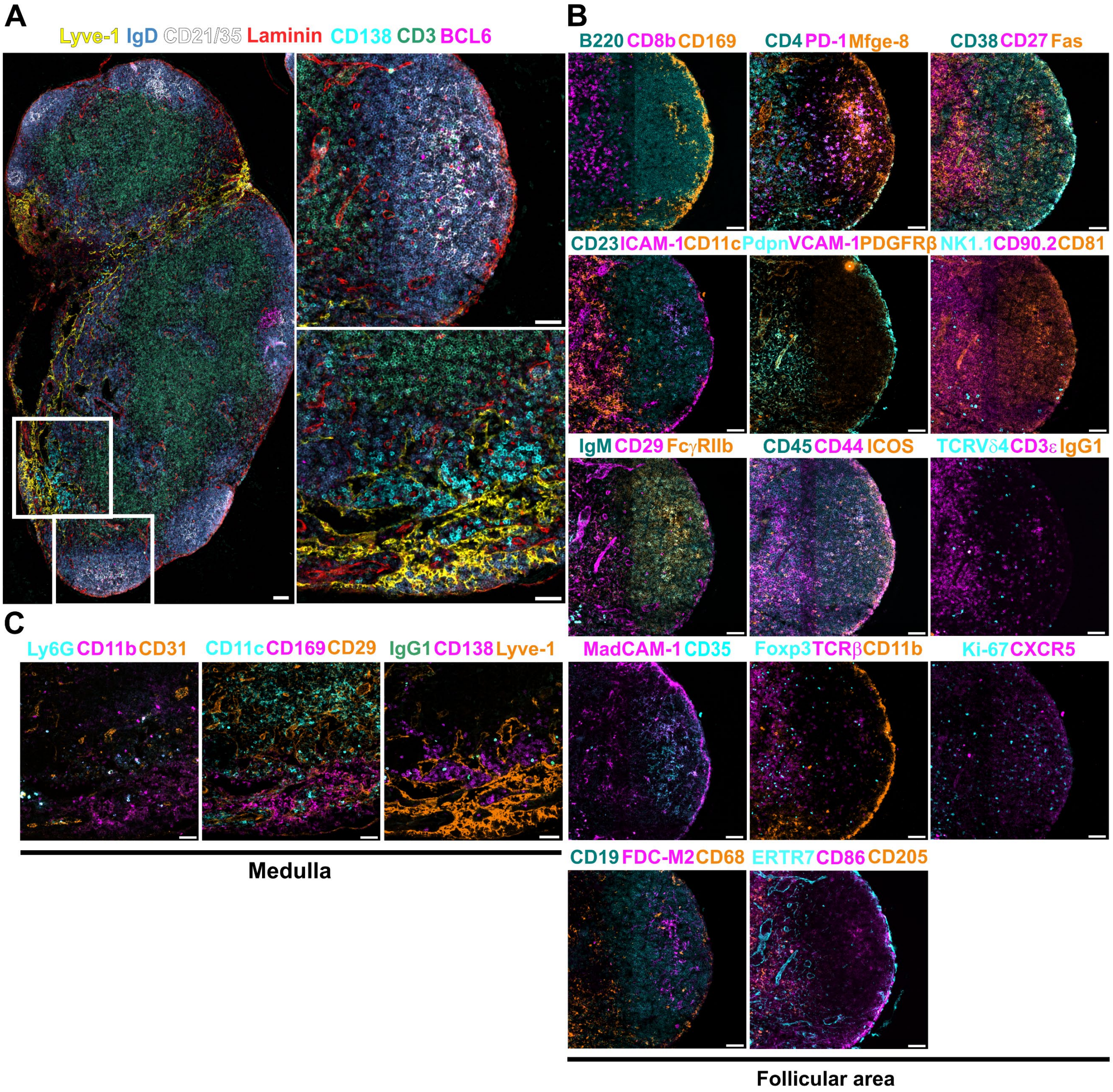

Figure S5. High-plex imaging of lymph nodes from a naive mouse. (**A**) A panoramic overview image displays six selected biomarkers in the entire LN. The white squares represent follicular and medullary areas of the LN, which are shown at higher magnification. (**B, C**) Representative photomicrographs of the 50-plex panel in a LN from a non-immunized. Three-color combination images displaying the different biomarkers in follicular (**B**) and medullary areas (**C**). Scale bars: 500 µm for the overview image in panel A (left), and 300 µm for individual photomicrographs in panels A (right), B, and C.

Figure S6

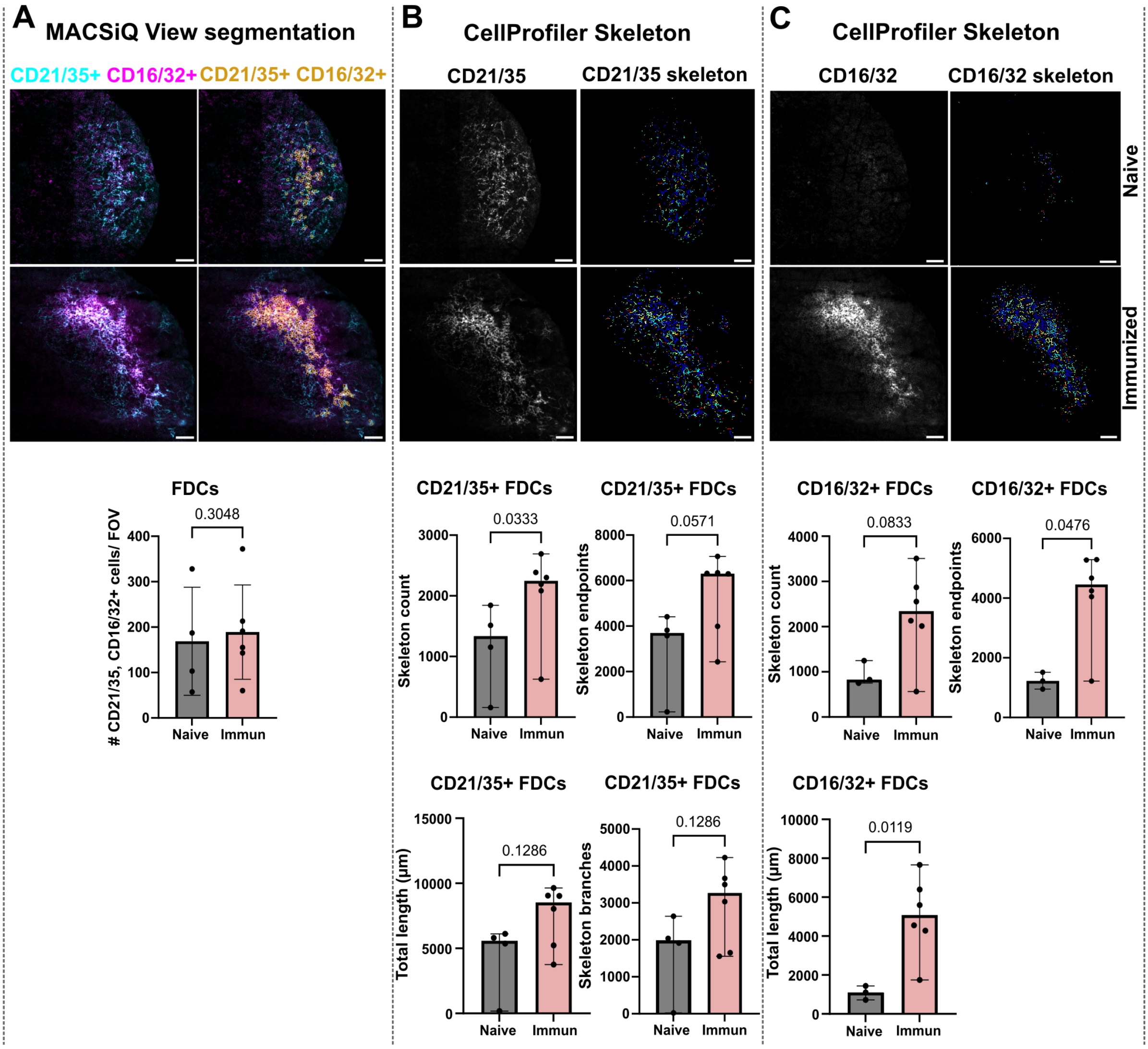

Figure S6. Morphological quantification of follicular dendritic cells using standard cell-segmentation or morphological skeleton analysis in the LN. (**A**) Identification of CD21/35 and CD16/32 double-positive cells (highlighted in yellow) using MACSiQ view software. The quantitative analysis of the number of double-positive cells is presented in the bar graph below. (**B, C**) Image analysis of CD21/35+ or CD16/32+ FDC networks using skeleton transformation in CellProfiler is displayed for comparison. The bar graphs below represent skeleton-derived parameters that reflect morphological changes in CD21/35+ (**B**) and CD16/32+ (**C**). FDC networks were quantified in individual FOVs (500 µm × 500 µm). Statistically significant *p* values ($p < 0.05$; one-sided *Mann-Whitney U test*). Scale bars: 300 µm. Abbreviations: CD, cluster of differentiation; GC, germinal center; FDC, follicular dendritic cell: FOV, field of view.
